# Cause-specific mortality among workers in asbestos mining and enrichment factories (Asbest Chrysotile Cohort Study) compared with the general population of Sverdlovsk Oblast, Russian Federation

**DOI:** 10.1186/s12995-025-00484-3

**Published:** 2025-11-06

**Authors:** Ann Olsson, Joachim Schüz, Igor Bukhtiyarov, Monika Moissonnier, Evgenia Ostroumova, Gilles Ferro, Eleonora Feletto, Graham Byrnes, Iraklii Tskhomariia, Kurt Straif, Tatiana Morozova, Hans Kromhout, Evgeny Kovalevskiy

**Affiliations:** 1 International Agency for Research on Cancer (IARC/WHO), Lyon, France; 2 Federal State Budgetary Scientific Institution “Izmerov Research Institute of Occupational Health” (IRIOH), Moscow, Russian Federation; 3 I.M. Sechenov First Moscow State Medical University (Sechenov University), Moscow, Russian Federation; 4 The Daffodil Centre, The University of Sydney, Cancer Council New South Wales, Woolloomooloo, Australia; 5 Institute for Risk Assessment Sciences, Utrecht University, Utrecht, The Netherlands

## Abstract

**Objectives:**

Complementing the previously published cohort-internal comparison we hereby compare the cause-specific mortality in workers of the Asbest Chrysotile Cohort study (ACC) with the general population of Sverdlovsk Oblast where the mine and factories are located.

**Methods:**

The ACC cohort database and the regional Federal State Statistic Service for the Sverdlovsk Oblast population were used. We calculated sex-specific standardized mortality ratios (SMRs) and 95% confidence intervals (CI) for the main ICD-10 disease groups and for selected cancer sites for the populations ≥ 15 years from 1980 to 2015. In relation to exposure, we applied the Sverdlovsk rates to the person-years in each exposure category to obtain expected numbers of lung cancer deaths. For comparing types of ACC workers (mine, enrichment factories, both), we calculated lung cancer SMRs in men by duration of employment (< 5 years, 5–14 years, and ≥ 15 years) and start of employment (before or after 1975) with miners as reference in each stratum.

**Results:**

Overall mortality of men in the ACC was reduced by 14%, mainly because of the most common cause of death, circulatory disease; this effect was much weaker in women. Elevated mortality was observed for both sexes from diseases of the digestive system (10–30%) and blood and blood forming organs (121–181%). Lung cancer mortality in men was increased from the 3rd quartile of chrysotile containing dust and in the highest quartile with SMR 1.30, 95% CI 1.07–1.57, while the increase in high exposed women was not reaching statistical significance (SMR 1.69, 95% CI 0.87–2.96). No increased SMRs were seen for laryngeal, stomach or ovarian cancers. Male factory workers first employed before 1975 had higher lung cancer mortality compared to miners, but not when employed after 1974.

**Conclusions:**

The observed excess in lung cancer confirms previous observations in the ACC. Risk management measures in the enrichment factories may have reduced the lung cancer risk to the level of the miners in recent decades.

## Introduction

The Russian Federation is the world’s largest country, and the Ural Mountains form the natural boundary between the continents of Europe and Asia. Sverdlovsk Oblast (an administrative division similar in size to a county or region), located in the Ural Federal District and inhabited by 4.2 million people in 2024, is rich in natural resources such as metals, minerals, marble, coal, and forests [1]. Asbest town in Sverdlovsk Oblast is named for its asbestos industry operating the world’s largest active open-pit chrysotile mine and its enrichment factories since 1896. The Asbest Chrysotile Cohort Study (ACC) is a historical cohort study of people employed by the Public Joint Stock Company (PJSC) Uralasbest in Asbest town [2]. The main cohort analysis comparing cohort members internally by individual cumulative exposure to dust and chrysotile fibres showed an increased risk of mesothelioma mortality with cumulative exposure to chrysotile asbestos from 40 fibers/cm^3^-years and in men increased lung cancer mortality with increasing cumulative exposure to chrysotile contained dust [3].

During the ACC follow-up between 1976 and 2015, mortality patterns and life expectancy have varied due to fluctuations in social, economic, and lifestyle circumstances despite the availability of free, universal health care to all Russian citizens. The most important factors contributing to the relatively low life expectancy in men (average life expectancy of 65 years in 2000) in the Russian Federation was a high mortality rate among working-age males from preventable causes such as alcohol, smoking, traffic accidents, and violent crimes [4, 5]. The standard retirement age until 2018 was 60 years for men and 55 years for women. However, an individual could be eligible for retirement from 50 years for men and 45 years for women if they had worked 10 years for men and 7.5 years for women in working conditions classified as harmful by the Special Federal Law No. 173-FZ “On Labor Pensions in the Russian Federation” Article 27 [6]. However, the opportunity to retire early was only mandatory in rare cases where there was a clear medical conclusion that continuing work in these specific conditions was impossible due to health reasons. In all other cases, the decision to retire early was made individually by the employee. Since earnings were better when working compared to the pension, most members of our cohort chose to continue working at their previous jobs.

Most of the ACC workers lived in Asbest town, i.e. nearby the mine and in proximity to the factories leading to environmental exposure to chrysotile containing dust. Therefore, it is of interest to compare the cohort with an external population. Complementary to the cohort-internal analyses already published [3], the primary objective of the present paper was to calculate sex-specific standardized mortality ratios (SMR) and relative standardized mortality ratios (rSMR) for main ICD-10 diagnostic groups and selected cancer sites comparing workers enrolled in the ACC with an external population, i.e. the population of Sverdlovsk Oblast. The rational was to compare the ACC with an external population and allow better comparison with Italian cohorts and the Qinghai chrysotile miner cohort in China [7, 8], and with the rSMR attempt to control for the healthy worker survival effect [9]. A secondary objective was to report additional results for the ACC workers on lung cancer mortality by cumulative dust exposure categories as applied in the main risk analyses, age-specific mortality rates, as well as comparing SMR for lung cancer for different groups of workers: miners, factory workers and those who worked in both while considering start of and duration of employment.

## Methods

The ACC includes 30,445 current and former employees (20,662 men, and 9,783 women) in the mine, all factories, auto-transport and external rail transportation departments, the central laboratory, and the explosives unit of the PJSC Uralasbest company, with at least one cumulative year of employment between 01/01/1975 and 31/12/2010 [3]. Cohort members were followed from 01/01/1976 through 31/12/2015. At the end of the follow-up, 52% of the cohort members were alive (9,972 men (48%), and 5,861 women (60%)), 36% were deceased (8,270 men (40%), 2,840 women (29%)), and 12% were censored (2,420 men (12%), 1,082 women (11%)). Cohort members were assigned annual average dust concentrations based on the job performed in each calendar year of their employment history at PJSC Uralasbest via a company-specific job-exposure matrix. The ACC job-exposure matrix was constructed from more than 90,000 stationary airborne dust measurements across workplaces in the factories (1951–2001) and the mine (1964–2001) [10]. Cumulative dust exposure was calculated by summing the average annual dust concentrations for all years of employment for each individual worker [2].

For the cohort members who died in Sverdlovsk Oblast, the cause of death was derived from the regional Civil Act Registration Office’ (ZAGS) electronic death certificates’ database via deterministic record linkage, complemented by manual searches including data from the Medical Information Analytical Centre (MIAC) [11]. ZAGS provided individual’s causes of death as original text that was coded according to ICD-10 by an experienced Russian physician working at IARC/WHO [11, 12]. Cohort members that left the oblast were censored at their last known date living in Sverdlovsk Oblast.

The ACC worker’s occupational history was based on company records, and vital status via record linkage followed up for mortality obtained from official death certificates of Sverdlovsk Oblast. Therefore, cohort enrolment and follow-up did not require individual contact with or consent from the study participants. The ACC was approved by the International Agency for Research on Cancer (IARC/WHO) Ethics Committee (IEC No. 12–22, September 2012). The IEC and an independent Scientific Advisory Board (see https://asbest-study.iarc.who.int/study-oversight/scientific-advisory-board) monitored the progress of the study on a regular basis.

Data on population size and mortality rates in Sverdlovsk Oblast were obtained from the Sverdlovsk regional territorial body of the Federal State Statistic Service of the Russian Federation. For mortality rate comparisons in the ACC with the rest of the Sverdlovsk Oblast, the numbers of deaths and person-years at risk of the cohort were subtracted from the respective strata of the Sverdlovsk Oblast population. Hereafter, the Sverdlovsk Oblast refers to the oblast excluding the deaths and person-years at risk from the ACC. Up to 1998 causes of death in Russia were coded with the Soviet system of disease classification corresponding to larger groups of diseases in ICD-9 (International Classification of Diseases, ninth revision) [13]. From 1999 onwards the codes were more detailed and based on the ICD-10. The re-coding from ICD-9 to ICD-10 was done in a previous project comparing mortality in Asbest city and Sverdlovsk Oblast where the authors noted that it was not possible to isolate some specific cancer types within the earlier coding systems (e.g., mesothelioma) [14].

### Statistical analysis

We included main ICD-10 diagnostic groups (chapters I Infectious and parasitic diseases (A00-B99), II Cancer (C00-C97), III Diseases of blood and blood forming organs (D50-D89), IV Endocrine, nutritional, metabolic diseases (E00-E90), V Mental and behavioral disorders (F00-F99), VI Diseases of nervous system G00-G99), IX Diseases of circulatory system (I00-I99), X Diseases of respiratory system (J00-J99), XI Diseases of digestive system (K00-K93), XIV Diseases of genitourinary system (N00-N99), XX All external causes (S00-Y98)), and selected cancer sites with large-enough numbers and available oblast data for the relevant time periods. Age-specific rates by 5-year age groups and 5-year periods for the population ≥ 15 years old were calculated in the oblast, separately for men and women. These rates were applied to the person-years in the ACC to calculate the expected number of deaths in the ACC. Then, the standardized mortality ratios (SMRs) were calculated using the ratio of observed number of deaths to the expected number of deaths in the ACC. As workers enrolled in the cohort had to work one cumulative year after 1 January 1975, there was a deficit in the mortality in the first years of follow-up. Therefore, the mortality of the first 4 years of the follow-up of the ACC (1976–1979) was omitted and the SMR calculations started in the year of 1980.

Official statistics on mortality rates were lacking for ovarian cancers for the period of 1980–1998, so we used the rates from the period of 1999–2015 and extrapolated to 1980–1998. Laryngeal cancer was analysed for men but not for women because there were no deaths from laryngeal cancer among the female workers in the cohort. Liver cancer was not included because the incidence varied over the follow-up time, and we only had oblast data for 6 years between 2002 and 2008.

To “control” for the healthy worker survival effect, we estimated relative standardized mortality ratios (rSMR) (ratio between the cause specific SMR and the overall SMR) [9].

To obtain the expected number of deaths in Sverdlovsk Oblast we applied the Sverdlovsk rate to the person-years in each exposure strata.

Crude age-specific rates for age categories of < 40 years, 10-years age groups, and 80+, were calculated for the period from 1980 to 2015 to compare the ACC with Sverdlovsk Oblast.

For comparing types of ACC workers, we calculated SMRs for lung cancer mortality in the ACC comparing the mortality in the factory workers (including the central laboratory workers) to the mortality in the miners (including workers in the external rail, auto transportation, and explosion units) and to those working in both by duration of employment (< 5 years, 5–14 years, and ≥ 15 years) and start of employment (before or after 1975). For each comparison, death rates in the mine workers were used as a reference category because exposures to dust in the mine is lower than in the factories due to the outdoor work location (open pit mine). Furthermore, due to the enrichment process, dust and fibre concentrations were generally higher in the factories [10]. This analysis was not done for women due to fewer cases of lung cancer and relatively few women working for long time periods in the mine.

The statistical package Stata 17 Standard Edition was used for all calculations.

## Results

Among men, the most frequent causes of death in the ACC were diseases of circulatory system (44%), external causes (20%), and cancer (19%), while in women circulatory system diseases (54%), were followed by cancer (19%), and then external causes (9%). The average age at death from cardiovascular diseases was 64 years for men and 70 for women, for cancer – 64 years for men and 63 for women, and for external causes – 48 years for men and 53 years for women.

**Table 1:** Standardized mortality ratios (SMR) and 95% confidence intervals (95% CI) and relative standardized mortality ratios (rSMR) in men and women aged 15 + years in the Asbest Chrysotile cohort study (ACC) compared to the corresponding populations in Sverdlovsk Oblast for main ICD-10 diagnostic groups and selected cancer types in the period from 1980 to 2015

Cause of death (ICD-10 codes)	ACC	ACC vs. Sverdlovsk Oblast			Relative standardized mortality ratios
MEN	Deaths observed	SMR	95% CI		rSMR
All-causes (A00-Z99)	8082 *(100%)*	0.86	0.84	0.88	
Infectious and parasitic diseases (A00-B99)	142 *(2%)*	0.48	0.40	0.56	0.55
Cancer (C00-C97)	1494 *(19%)*	1.05	1.00	1.10	1.22
Stomach (C16)	178	0.94	0.81	1.09	1.10
Colorectal (C18-C21)	134	0.95	0.80	1.13	1.11
Larynx (C32)	45	0.98	0.72	1.31	1.14
Trachea, bronchus, lung (C33-C34)	556	1.20	1.10	1.30	1.39
Prostate (C61)	58	0.97	0.73	1.25	1.13
Urinary tract (C64-68)	106	1.13	0.93	1.37	1.32
Haematological malignancies (C981-96)	59	1.10	0.84	1.42	1.28
Diseases of blood and blood forming organs (D50-D89)	9 *(0.1%)*	2.21	1.01	4.20	2.58
Endocrine, nutritional, metabolic diseases (E00-E90)	22 *(0.1%)*	0.76	0.47	1.14	0.88
Mental and behavioural disorders (F00-F99)	9 *(0.1%)*	0.18	0.08	0.34	0.21
Diseases of nervous system (G00-G99)	73 *(1%)*	0.91	0.71	1.14	1.06
Diseases of circulatory system (I00-I99)	3562 *(44%)*	0.89	0.86	0.92	1.04
Diseases of respiratory system (J00-J99)	322 *(4%)*	0.59	0.53	0.66	0.69
Diseases of digestive system (K00-K93)	429 *(5%)*	1.10	1.00	1.21	1.28
Diseases of genitourinary system (N00-N99)	63 *(1%)*	1.15	0.89	1.48	1.34
All external causes (S00-Y98)	1604 *(20%)*	0.73	0.69	0.76	0.84
WOMEN					
All-causes (A00-Z99)	2801 *(100%)*	0.98	0.94	1.02	
Infectious and parasitic diseases (A00-B99)	28 *(1%)*	0.93	0.62	1.34	0.95
Cancer (C00-C97)	526 *(19%)*	1.01	0.93	1.10	1.04
Stomach (C16)	66	1.08	0.83	1.37	1.10
Colorectal (C18-21)	76	0.94	0.74	1.18	0.97
Trachea, bronchus, lung (C33-34)	41	1.35	0.97	1.83	1.37
Breast (C50)	98	1.07	0.87	1.31	1.10
Cervix uteri (C53)	12	0.57	0.29	0.99	0.58
Ovary (C56)	34	0.96	0.67	1.34	0.98
Urinary tract (C64-68)	14	0.78	0.43	1.32	0.80
Haematological malignancies (C81-96)	33	1.36	0.94	1.91	1.39
Diseases of blood and blood forming organs (D50-D89)	6 *(0.2%)*	2.81	1.03	6.12	2.87
Endocrine, nutritional, metabolic diseases (E00-E90)	29 *(1%)*	1.01	0.68	1.46	1.04
Mental and behavioural disorders (F00-F99)	3 *(0.1%)*	0.48	0.10	1.40	0.49
Diseases of nervous system (G00-G99)	25 *(1%)*	0.95	0.62	1.41	0.97
Diseases of circulatory system(I00-I99)	1503 *(54%)*	0.93	0.88	0.98	0.95
Diseases of respiratory system (J00-J99)	65 *(2%)*	0.78	0.60	0.99	0.80
Diseases of digestive system (K00-K93)	159 *(6%)*	1.30	1.10	1.51	1.32
Diseases of genitourinary system (N00-N99)	40 *(1%)*	1.50	1.07	2.05	1.54
All external causes (S00-Y98)	240 *(9%)*	0.80	0.70	0.91	0.82

Table 1 shows the sex-specific SMRs and rSMRs by the main ICD-10 groups and selected cancer sites. The mortality in the ACC male workers compared with the male population of Sverdlovsk Oblast were lower for all causes of death, infectious and parasitic diseases, mental and behavioral disorders, diseases of circulatory system, diseases of respiratory system, and all external causes. In contrast, the ACC male workers had higher mortality from diseases of blood and blood-forming organs other than cancer, diseases of the digestive system, and cancer driven by lung cancer. Indeed, the analyses of selected cancer sites showed a 20% increased mortality from lung cancer compared with Sverdlovsk Oblast.

The rSMRs in men were 17–19 units higher compared to the SMR, indicating that a “healthy worker survival effect” was present.

The mortality comparison between the ACC female workers and female population of Sverdlovsk Oblast showed lower mortality in the cohort for diseases of circulatory system, diseases of respiratory system and all external causes, but increased mortality for diseases of blood and blood-forming organs other than cancer, diseases of digestive system, and diseases of genitourinary system.

The analysis of site-specific cancer mortality showed a reduced SMR for cervical cancer, and somewhat elevated SMRs for lung cancer and hematological malignancies.

The rSMRs in women were only slightly higher, i.e. 2–3 units, compared to the standard SMR, indicating that the health profiles were similar in the cohort and Sverdlovsk Oblast.

**Table 2:** Standardized mortality ratios (SMR) for lung cancer in men and women aged 15 + years in the Asbest Chrysotile cohort study (ACC) compared to the corresponding populations in Sverdlovsk Oblast by Chrysotile asbestos categories in ACC, from 1980 to 2015

MEN	ACC	ACC vs. Sverdlovsk Oblast		
Dust category mg/m^3^-years	Lung cancer deaths observed	SMR	95% CI	
0*	3	0.52	0.11	1.52
> 0–20	89	1.02	0.82	1.25
> 20–65	155	1.11	0.94	1.30
> 65–150	209	1.26	1.09	1.44
> 150	108	1.30	1.07	1.57
**WOMEN**				
0*	-		-	-
> 0–20	7	1.36	0.55	2.79
> 20–65	11	1.20	0.60	2.15
> 65–150	11	1.05	0.52	1.88
> 150	12	1.69	0.87	2.96

* A 5-year lag time was applied. Thus, some workers ended up with no occupational exposure to dust although all workers had at least some occupational exposure to dust

Table 2 shows SMRs for lung cancer in relation to categories of cumulative exposure to dust, again comparing the ACC rates with those of Sverdlovsk Oblast. The analysis of cumulative dust exposure applied a time-dependent 5-year lag of cumulative dust for each individual worker, as was done in the main analysis [3]. When the lag time is applied, there are workers who end up with no occupational exposure because the observation time at risk is shorter than the lag time (e.g., with a 5-year lag, a worker who died less than 5 years after the first occupational exposure). This group is shown for completeness, as all workers in this analysis had at least some occupational exposure. The lowest exposure category (>0–20 mg/m^3^-years cumulative dust) shows almost identical lung cancer mortality in men across the two populations, and increased lung cancer mortality in the two highest exposure categories (>65 mg/m^3^-years). In women in the ACC the lung cancer mortality varied in a U-shape manner in comparison to Sverdlovsk Oblast, but with wide confidence intervals.

**Fig. 1:**
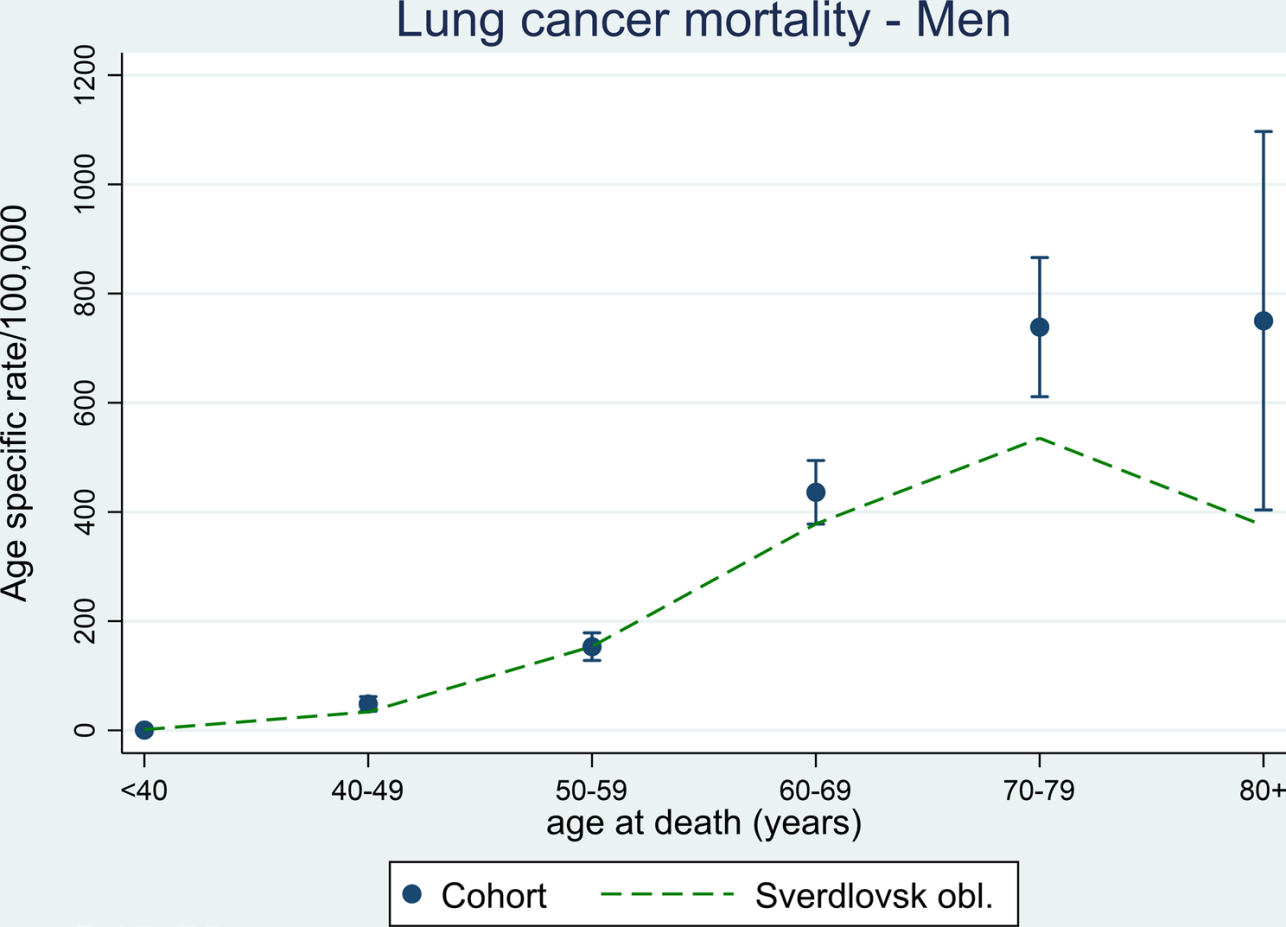
Age-specific mortality rates for lung cancer among men in the Asbest Chrysotile Cohort study with 95% confidence intervals and the population in Sverdlovsk Oblast during the period 1980–2015

Figure 1 displays lung cancer mortality notably higher in men in the ACC above the age of 60 years compared to the age-specific rates in Sverdlovsk Oblast. The rate for Sverdlovsk Oblast appears to decrease beyond the age of 70 years, while it does not decrease in the ACC. Confidence intervals are however wide for the age group 80 years and above.

**Fig. 2:**
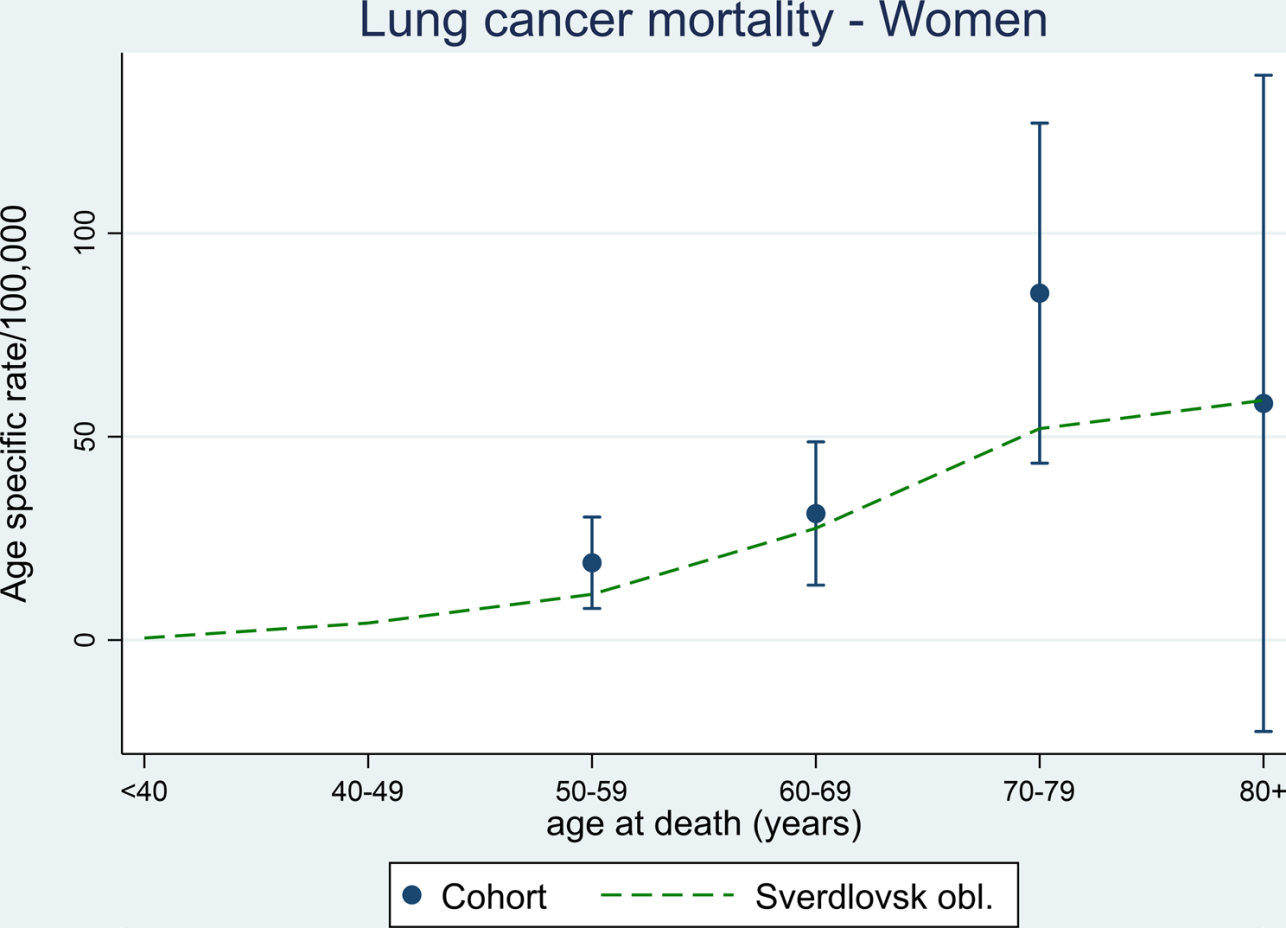
Age-specific mortality rates for lung cancer among women in the Asbest Chrysotile Cohort study with 95% confidence intervals and the population in Sverdlovsk Oblast during the period 1980–2015

The lung cancer mortality pattern in women in Fig. 2 shows lower rates than men overall, but with a modest peak in women in the ACC at around 70 years. Unlike the men, no noticeable decline in lung cancer mortality is seen in the female populations of Sverdlovsk Oblast beyond the age of 70 years.

**Table 3:** Internal comparison of standardised mortality ratios (SMR) and 95% confidence intervals (CI) for lung cancer mortality 1976–2015 in male miners, factory workers, and those having worked both in the mine and the factories in the Asbest Chrysotile cohort study by duration of employment and start of employment. The miners are the reference category (in bold) in all strata

LUNG CANCER MORTALITY MEN			Duration of employment					
			less 5 years		5–14 years		15 years and more	
			N workers/ deaths	SMR	N workers/ deaths	SMR	N workers/ deaths	SMR
start of employment	before 1975	mine	423/6	**1.00**	861/30	**1.00**	3,429/183	**1.00**
		factory	157/3	1.66 (0.34–4.86)	399/25	1.73 (1.12–2.55)	1,093/74	1.39 (1.09–1.75)
		both	330/10	1.77 (0.85–3.25)	819/32	1.03 (0.71–1.46)	1,603/99	1.19 (0.97–1.45)
	1975+	mine	3,642/26	**1.00**	2,283/26	**1.00**	1,346/18	**1.00**
		factory	1,056/6	0.86 (0.32–1.87)	752/4	0.56 (0.15–1.44)	267/4	1.43 (0.39–3.66)
		both	1034/9	1.62 (0.74–3.07)	804/7	1.10 (0.44–2.27)	364/2	0.80 (0.10–2.89)

Table 3 shows SMRs for lung cancer mortality in ACC male workers by type of work (mine, factory, both) and by start and duration of employment between 1976 and 2015. Male factory workers who started working before 1975 and worked for more than 5 years had a significantly higher lung cancer mortality compared to the male workers in the mine. Lung cancer SMR estimates for male workers who started working after 1974 were not significantly different by type of work.

## Discussion

We report sex-specific SMRs and rSMRs for the main ICD-10 groups and selected cancers, and SMRs for lung cancer mortality by cumulative exposure to dust in the ACC compared to the population of Sverdlovsk Oblast [14]. This analysis is complementary to the cohort-internal mortality risk analyses by cumulative exposure to dust and fibers, which were published in 2024 [3]. Mortality overall and from circulatory and respiratory diseases, and external causes were lower in the ACC men and women compared to the population of Sverdlovsk Oblast. In contrast, the ACC men and women experienced increased mortality for the diseases of digestive system and diseases of blood and blood forming organs other than cancer, and for diseases of genitourinary system in women. All cancers, and especially lung cancer SMRs in the ACC men were elevated compared to Sverdlovsk Oblast male population. At a closer look, the increased SMRs for lung cancer were present in the two upper dust categories (>65 mg/m^3^-years). Lung cancer SMR in the ACC women was elevated but not at the level of statistical significance, while mortality from cervical cancer was decreased. Mortality from other causes were similar in ACC and Sverdlovsk Oblast.

Lower mortality from all-causes and specifically circulatory and respiratory diseases in men and women in the ACC compared to the population of Sverdlovsk Oblast is most likely explained by a healthy worker survivor effect, i.e. cohort members had a better health because workers are employed at PJSC Uralasbest considering their initial health status at hire. Women applying for a job also underwent bacteriological (for flora), cytological (for atypical cells), and ultrasound examinations by an obstetrician-gynecologist. In addition, workers underwent obligatory and regular health checks throughout their career. In men, the healthy worker survivor effect was confirmed by calculating the rSMRs. The healthy worker survivor effect was not noticeable in the female workers. This may be explained by women being more prone to use available health services and generally being more attentive to their health in terms of less smoking and drinking. This aligns with the findings of our main risk analysis of an inverse association between all-cause mortality with cumulative dust exposure only in men [3].

Both men and women in the ACC had an increased mortality from diseases of the digestive system like the findings when comparing the mortality in Asbest town with Sverdlovsk Oblast between 1997 and 2010 [14]. The leading cause-of-death in this category in the ACC was liver cirrhosis, other and unspecified (ICD-10 code: K74.6), representing 25% of the deaths from diseases of digestive system among men and 32% among women, but we did not have the corresponding detailed data from Sverdlovsk Oblast. To our knowledge only few asbestos cohorts have analysed diseases of the digestive system other than cancer or cirrhosis, with one worker in a Greek chrysotile cohort reported deceased of liver cirrhosis [15], and the results from the Balangero cohort (Italy) of chrysotile asbestos workers showed an increased mortality from liver cirrhosis with SMRs of ~ 300 [16, 17]. For the latter study, authors referred to the high alcohol consumption in the cohort as the likely cause of the cirrhosis. In ACC, it is unlikely that high alcohol consumption is the sole cause for the increased SMR from liver cirrhosis because we did not see respective increases in mortality from other alcohol-related causes of death such as external causes, stomach or colorectal cancers. In addition, cohort members likely had fewer opportunities for regular alcohol consumption compared to the general male population due to mandatory pre-shift alcohol testing in many occupations in the cohort. In view that not only ACC but the Asbest town showed increased mortality from diseases of the digestive system when compared to the mortality pattern in Sverdlovsk Oblast [14] one may suspect an environmental cause.

The SMRs were elevated in both men and women for diseases of blood and blood forming organs other than cancers based on nine cases in men and six in women over the follow-up period between 1980 and 2015. To our knowledge this result has not been reported previously and should be interpreted with caution due to the small numbers. The diseases within this group were a mix and showed no clear patterns. However, iron deficiency anemia is secondary to blood loss (chronic), i.e. ICD-10 code: D50.0 (*n* = 5), and could reflect underdiagnosed cancers [18].

An increased mortality from genitourinary diseases of about 50% was observed in the ACC women based on 50 deaths. An Italian pooled study of asbestos workers’ cohorts also showed an elevated SMR of 30% [8]. The most frequent genitourinary diseases in the ACC women were unspecified chronic tubulo-interstitial nephritis (ICD-10 code: N11.9) representing 40% of the genitourinary disease deaths, and unspecified chronic nephritic syndrome (ICD-10 code: N03.9) − 18%. Tubulointerstitial nephritis has multiple etiologies, including drug-related (e.g. beta-lactam antibiotics, non-steroidal anti-inflammatory (NSAID)), infectious, systemic, autoimmune, genetic, and idiopathic [19], but all these would be rather speculative explanations for our cohort. We also found no experimental studies that discuss asbestos exposure in relation to diseases other than cancer either of the digestive system, blood and blood forming organs, or genitourinary organs.

The somewhat reduced mortality from cervical cancer is likely due to the enhanced health checks within the PJSC Uralasbest company compared to the general population.

The increased mortality from all cancers in men in the ACC is mainly driven by the increased lung cancer mortality. The lung cancer SMR for chrysotile asbestos >150 mg/m^3^-years of 1.30, 95% CI 1.07–1.57 aligns with the main risk analyses showing relative risk (RR) = 1.40, 95% CI 1.03–1.90 for the highest exposure category when compared to the lowest (>0–20 mg/m^3^-years) category [3]. Liddell et al. also reported increased lung cancer risk from the 1891–1920 birth cohort of Quebec chrysotile miners and millers (SMR = 1.37) including a not formally tested exposure-response relationship [20]. Likewise, results from the Chinese chrysotile miner cohort disclose an internal exposure-response relationship and an overall lung cancer SMR of 4.69, 95% CI 3.61–6.09 [7]. An update of the Balangero cohort in Italy showed an overall lung cancer SMR of 1.14, 95% CI 0.81–1.55 among the chrysotile asbestos miners with an indicative exposure-response for cumulative exposure to fibers (f/mL-y) [21]. Likewise, a small Greek chrysotile cohort showed increased mortality from lung cancer (SMR = 1.71, 95% CI 0.98–2.78) [15]. A pool of Italian asbestos workers cohorts showed lung cancer SMR of 1.28, 95% CI 1.24–1.32 in men and 1.26, 95% CI 1.02–1.53 in women [8].

So why do we observe a stronger association between dust and lung cancer in men compared to women in ACC? Women were exposed to higher median cumulative exposure to dust (49.4 mg/m^3^-years) than men (31.4 mg/m^3^-years) as they were more often employed in factories with higher exposure concentrations than in the mine, and women worked on average longer (median 10.9 years) compared to men (median 9.3 years) [10]. From the literature we know that smoking exerts a more than additive joint effect with occupational exposure to asbestos, which may be an explanation [22]. A survey in 2017 among current and former employees of the PJSC Uralasbest showed high prevalence of smoking among men (66%), while very low prevalence of smoking among women (9%) [23]. The difference between men and women in smoking rates will have been larger among earlier employed members of the cohort. Co-exposures may also have affected men more than women and played a role in the difference in risk between men and women. 46% of workers (*n* = 14,133) had at least one co-exposure listed in their company records. Respirable crystalline silica was assumed to affect all workers, while welding was more frequent in the factories and diesel engine exhaust was more frequent in the mine.

Comparison of workers having worked only in the mine, only in the factories, and in both by the start of employment and duration of employment showed higher lung cancer mortality among factory workers when hired before 1975, while in workers hired after 1974 there was no significant difference in risk by type of work. It is likely the result of risk management measures more effective in the enrichment factories than in the mine [10].

Strengths of the ACC include the large size of the cohort (*n* = 30,445), a substantial proportion of female workers (32%) to investigate sex-specific risks, long period of mortality follow-up (1976–2015), and availability of original text on cause of death from death certificates of all deceased cohort members from two official sources, ZAGS and MIAC of the Ministry of Health of the Sverdlovsk Oblast, complementing each other. A quality control exercise comparing cause-of-death information for 5,463 deaths registered between 2002 and 2015 between the two sources showed an agreement of 97.9% at the top-level ICD-10 codes. For 1,009 cancer deaths, the interrater agreement between the ICD-10 coding performed by IARC/WHO using ZAGS information and the MIAC coding of deaths from malignant neoplasms was 96.4% [12]. In short, we assume good reliability of causes-of-death coding for the ACC as it was performed by the same person at one point in time.

As a study limitation we acknowledge potential heterogeneity and lack of detailed data from the region over the follow-up time, which could affect the comparisons of mortality rates in our cohort with the data from Sverdlovsk Oblast. The relatively low average age at death observed in both populations during the study period may have masked increased mortality of cancers and other end points occurring mostly at old ages. We have previously conducted cohort-internal analyses for total mortality and lung cancer mortality on the “inception cohort”, i.e. workers first employed 01.01.1975 or later. While the results showed similar patterns confidence intervals were much wider, i.e. for dust 150 + mg/m3-years total mortality in men RR 0.55, 95% CI 0.28–1.11 based on 8 cases, and in women RR 0.70, 95% CI 0.31–1.58 based on 6 cases. The corresponding results for lung cancer mortality in men were RR 2.21, 95% CI 0.54–9.14 based on 2 cases, and in women for the second highest exposure category (65–150 mg/m3-years) RR 1.08, 95% CI 0.10-12.04 based on 1 case. The low numbers of death together with most study participants in the inception cohort attaining low exposure levels resulted in lack of statistical power to examine if trends by stratification of time of enrolment in the ACC were different.

In conclusion, despite their occupational exposures to dust and chrysotile asbestos, all-cause mortality in the ACC was lower than in the Sverdlovsk Oblast, especially in men. This confirms the assumed healthy worker survivor effect from the previous cohort analysis [3] most likely caused by hiring particularly healthy people and the regular medical monitoring of workers working in hazardous conditions. Lung cancer SMRs were significantly increased in men with occupational cumulative exposure to dust containing chrysotile asbestos fibers exceeding 65mg/m^3^-years. Lung cancer SMRs in women were elevated to a similar extent in a U-shape manner and with all confidence intervals including 1. No increased SMRs were seen for laryngeal, stomach or ovarian cancers. Analyses by type of work showed that before 1975 the male factory workers experienced higher lung cancer mortality than other male workers, while in workers employed after 1974 the risks were comparable, presumably because of successful implementation of risk management measures in the enrichment factories. Continued follow up is planned and warranted. The increased mortality from diseases of the digestive system and blood and blood forming organs other than cancer remain without a credible explanation.

## Data Availability

Raw data cannot be made publicly available according to the data confidentiality legislation of the Russian Federation.
